# Home Nursing for Insured Persons

**Published:** 1922-11

**Authors:** F. T. West

**Affiliations:** Clerk to the Cheshire Insurance Committee. 28, Nicholas Street, Chester


### CORRESPONDENCE.

[Correspondence on all subjects is invited, but we cannot be responsible for the opinions expressed by our correspondents, who must give name and address as a guarantee of good faith, but not necessarily for publication. Correspondents are reminded that conciseness greatly facilitates early insertion.]

### Home Nursing for Insured Persons.

*To the Editor of* THE HOSPITAL AND HEALTH REVIEW.

SIR: I am directed by the Cheshire Insurance Committee to request that you will kindly permit the use of your valuable paper for the expression of its opinions and the conclusions at which it has arrived in connection with the domiciliary nursing of insured persons. For some considerable time its attention has been concentrated on this question, and during the current year an attempt was made to promote a scheme in the county of Cheshire.

Briefly, the scheme provided for the services of nurses being made available to all insured persons entitled to medical benefit, irrespective of their right to participate in additional benefits out of "Valuation Surpluses" or otherwise; the cost of the scheme to be borne by contributions from Approved Societies. It was further proposed that the cost of the administration of the scheme should be borne by the Insurance Committee, and should not be a charge upon the nursing services. It was proposed to utilise all existing nursing agencies in the carrying out of the scheme.

The result of the Committee's inquiries as to whether the Approved Societies having members in the area were prepared to contribute to such a scheme has not been sufficiently satisfactory to justify the Insurance Committee in proceeding to put the scheme into operation. In many cases the Approved Society stated that it would be prepared to contribute to the scheme, but inasmuch as in other parts of the country such schemes were not in operation, the insured persons in Cheshire would thereby have an undue advantage over their members in other parts of the country; they could not, therefore, see their way to contribute. This appears to be the main difficulty which confronts Approved Societies in the matter, and points to the necessity of inaugurating a system of nursing for insured persons, national in its character and application, as a solution of the problem.

In their report upon this matter, the Association of Approved Societies, through their Nursing Subcommittee, so long ago as 1913, stated that "ever since the Insurance Bill was introduced into Parliament discussions have taken place upon the question of supplementing medical and sickness benefits by a scheme of trained nursing, and any scheme adopted must be a uniform one, and must be started upon a more permanent foundation than would be afforded by any local scheme adopted by one or more individual Societies." In 1914 Parliament provided a substantial grant in aid for this purpose, in respect of which, when introducing the matter, the present Prime Minister stated that "the Insurance Act has helped to make it clear that any system of doctoring is hopelessly inefficient which is not supplemented by a good system of nursing." He further stated "there are Voluntary Associations throughout the country doing admirable work, but they are inadequately financed." Certain Approved Societies have already made arrangements with the Queen Victoria Jubilee Institute for Nurses for the provision of nursing to the members of such Societies, but this is not in any sense a national scheme.

The subject is of vital importance to the health of the nation. The efforts of the medical practitioner, supplemented by the pharmacist, are often rendered fruitless by lack of trained nursing, especially when the patient is a woman unable to obtain any help from outside, and forced to rely upon her husband or young children to give effect to the doctor's instructions in the preparation of her food, medicine or otherwise. Medical men acknowledge that in a high percentage of cases the period of disability is unnecessarily prolonged by reason of the absence of the services of a trained nurse. As already expressed by a well-known medical man, the ultimate success of National Health Insurance must rest upon the tripod—doctor, chemist and nurse. The doctor and chemist have been provided, and it is essential to the complete success of the scheme that national nursing should immediately be forthcoming. It is held that it should be an integral part of medical benefit to which an insured person is entitled under the National Insurance Acts. Any partial scheme relating to the members of only certain Approved Societies involves the Societies, the nursing associations, and all concerned in additional work of discrimination between those entitled and others not so fortunate. It draws a distinction between insured persons which should not exist, having regard to the fact that no variations in rates of contributions under the National Insurance Acts is contingent thereupon.

It is hoped that the expression of these views through your columns may concentrate public opinion upon a matter which has become pressing, and thereby to bring pressure to bear upon Government to go forward with the inauguration of a national system of nursing, which was considered so urgently necessary and desirable in 1914.

I am, Sir,
Your obedient Servant,
F. T. WEST,
Clerk to the Cheshire Insurance Committee.
28, Nicholas Street, Chester.

---

Dr. F. J. Waldo, H.M. coroner of the City of London and Southwark, has recently completed 21 years' service in that position.

For the third time in 20 years the Medical Officer for East Barnet, Dr. Elam, has reported a clean bill of health in the East Barnet Valley district. He is to be presented with a pair of white gloves by the District Council.

For taking her baby home to London in a crowded train after being told that the child had diphtheria, a Deptford woman who had come to Ramsgate for a month's holiday was fined £5. The magistrate said that the full penalty was quite inadequate for such a serious offence.

